# 2-Nitro­anilinium bromide

**DOI:** 10.1107/S1600536811041948

**Published:** 2011-10-29

**Authors:** R. Anitha, S. Athimoolam, S. Asath Bahadur, M. Gunasekaran

**Affiliations:** a Department of Physics, Anna University of Technology Tirunelveli, Tirunelveli 627 007, India; b Department of Physics, University College of Engineering Nagercoil, Anna University of Technology Tirunelveli, Nagercoil 629 004, India; c Department of Physics, Kalasalingam University, Anand Nagar, Krishnan Koil 626 190, India

## Abstract

The title compound, C_6_H_7_N_2_O_2_^+^·Br^−^, is isomorphous with 2-nitro­anilinium chloride and contains an characteristic intra­molecular N—H⋯O hydrogen bond, forming an *S*(6) motif. Inter­molecular N—H⋯Br hydrogen bonds occur in the crystal structure. Two zigzag chains of *C*_2_^1^(4) motifs extend along the *b-*axis direction. These primary chain motifs inter­sect like a double helix structure, leading to *R*_6_^3^(12) ring motifs, which are arranged in tandem along the *b* axis. Hence, hydro­philic layers are generated at *z* = 1/4 and 3/4, which are sandwiched between alternate hydro­phobic layers across *z* = 0 and 1/2.

## Related literature

For related structures, see: Herbstein (1965 ▶); Dhaneshwar *et al.* (1978 ▶); Saminathan & Sivakumar (2007 ▶); Ploug-Sørensen & Andersen (1983) ▶. For hydrogen-bonding motifs, see: Bernstein *et al.* (1995 ▶); Desiraju (1989 ▶).
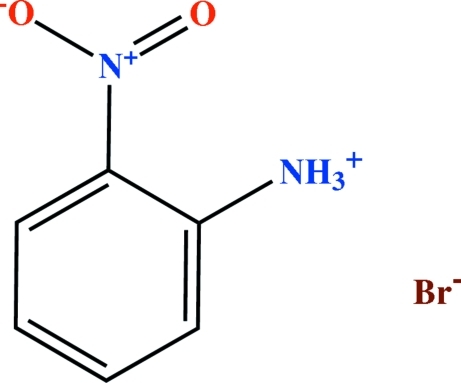


## Experimental

### 

#### Crystal data

C_6_H_7_N_2_O_2_^+^·Br^−^
*M* *_r_* = 219.05
Orthorhombic, 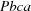

*a* = 8.0268 (8) Å
*b* = 8.1242 (7) Å
*c* = 23.7912 (19) Å
*V* = 1551.5 (2) Å^3^
*Z* = 8
Mo *K*α radiation
μ = 5.25 mm^−1^
*T* = 293 K
0.21 × 0.19 × 0.17 mm

#### Data collection

Bruker SMART APEX CCD area-detector diffractometer
12000 measured reflections
1372 independent reflections
1107 reflections with *I* > 2σ(*I*)
*R*_int_ = 0.085

#### Refinement

*R*[*F* ^2^ > 2σ(*F* ^2^)] = 0.031
*wR*(*F* ^2^) = 0.076
*S* = 0.96
1372 reflections
112 parameters
H atoms treated by a mixture of independent and constrained refinement
Δρ_max_ = 0.74 e Å^−3^
Δρ_min_ = −0.38 e Å^−3^

### 

Data collection: *SMART* (Bruker, 2001 ▶); cell refinement: *SAINT* (Bruker, 2001 ▶); data reduction: *SAINT*; program(s) used to solve structure: *SHELXTL/PC* (Sheldrick, 2008 ▶); program(s) used to refine structure: *SHELXTL/PC*; molecular graphics: *PLATON* (Spek, 2009 ▶); software used to prepare material for publication: *SHELXTL/PC*.

## Supplementary Material

Crystal structure: contains datablock(s) global, I. DOI: 10.1107/S1600536811041948/ff2032sup1.cif

Structure factors: contains datablock(s) I. DOI: 10.1107/S1600536811041948/ff2032Isup2.hkl

Supplementary material file. DOI: 10.1107/S1600536811041948/ff2032Isup3.cml

Additional supplementary materials:  crystallographic information; 3D view; checkCIF report

## Figures and Tables

**Table 1:** Hydrogen-bond geometry (Å, °)

*D*—H⋯*A*	*D*—H	H⋯*A*	*D*⋯*A*	*D*—H⋯*A*
N2—H1*N*⋯O1	0.79 (3)	2.32 (3)	2.702 (4)	111 (3)
N2—H1*N*⋯Br1^i^	0.79 (3)	2.70 (4)	3.291 (3)	133 (3)
N2—H2*N*⋯Br1^ii^	0.93 (6)	2.29 (6)	3.197 (3)	165 (4)
N2—H3*N*⋯Br1	1.02 (4)	2.26 (4)	3.284 (3)	176 (3)

Symmetry codes: (i) 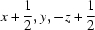; (ii) 
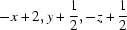
.

**Fig. 1.**
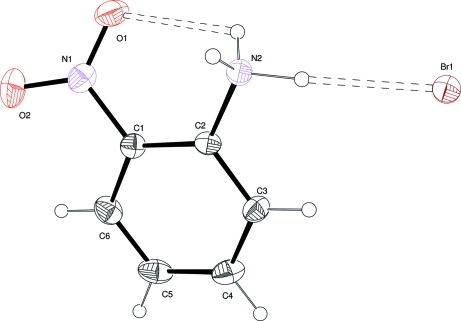
The molecular structure of the title compound (I) with the numbering scheme for the atoms and 50% probability displacement ellipsoids. H bonds are drawn as dashed lines.

**Fig. 2.**
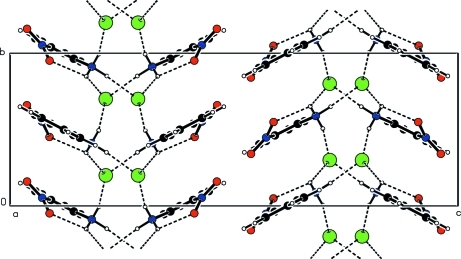
Packing diagram of the molecules viewed down the a-axis. H bonds are drawn as dashed lines.

## supplementary crystallographic information

### Comment

Intermolecular forces play an essential role in the formation of supramolecular systems which are useful for definite social applications. In which, the phenomenon of hydrogen bond has its importance in the areas of molecular recognition, crystal engineering research and supramolecular chemistry. Their strength and directionality is responsible for crystal packing and entire molecular arrays (Desiraju, 1989). 2-nitroaniline extists in three phases, *viz*., α- & β-polymorphs (Herbstein, 1965) & γ-polymorph (Dhaneshwar *et al.*, 1978). As a special attention, a non-proton transfer adduct of 2-nitroaniline with picric acid as 2:1 complex is reported by Saminathan & Sivakumar, 2007. Based on the above fact, we are interested on the specificity of recognition of nitroaniline with other inorganic/organic acids. Hence, the present work is attempted here.

The asymmetric part of the title compound, (I), contains one 2-nitroanilinium cation and a bromide anion (Fig. 1). The title compound is an isomorphous of 2-nitroanilinium chloride reported by Ploug-Sørensen & Andersen, 1983. There is only a quantitative change in the crystallographic parameters owing to the size of the anion; the unit cell volume in (I) is about 67 Å^3^ larger than that of the chloride salt (Ploug-Sørensen & Andersen, 1983). This present study was undertaken to investigate the hydrogen-bonding interactions with the concept of graph-set motifs, aggregation patterns and crystalline packing of the molecules. The protonation of the N site of the cation is evident from the elongated C—N bond distance. The plane of the nitro group (–NO_2_) is twisted out from the plane of the aromatic ring with an angle of 26.9 (2)°. Especially, the O atom which is involved in the intramolecular hydrogen bond is moved more away from the aromatic plane (0.565 (3) Å) than that of the other O atom (0.377 (4) Å) which is not participating in any hydrogen bonding interaction.

As nitroanilines have both donor (amine) and acceptor (nitro) sites for hydrogen bonding interactions, they have proved to be versatile reagents for structure extension by linear (chain C motifs) and cyclic (ring *R* motifs) hydrogen-bonding associations. In the present crystal structure, the molecular aggregations are stabilized through intricate three dimensional hydrogen bonding network (Fig. 2; Table 1). A characteristic intramolecular N—H···O hydrogen bond, forming an S(6) motif, is observed in the cation (Fig. 1). The other intermolecular hydrogen bonds are only N—H···Br type. Two zigzag chains of C_2_^1^(4)motifs are extending along *b*-axis of the unit cell. These primary chain motifs intersect like a double helix structure leading to ring *R*_6_^3^(12) motifs. These ring motifs are arranged in tandem along *b*-axis. Hence, hydrophilic layers are generated at *z* = 1/4 and 3/4 which are sandwiched between alternate hydrophobic layers across *z* = 0 and 1/2.

### Experimental

The title compound was crystallized from an aqueous mixture containing 2-nitroaniline and hydrobromic acid in the stoichiometric ratio of 1:1 at room temperature by slow evaporation technique.

### Refinement

All the H atoms except the atoms involved in hydrogen bonds were positioned geometrically and refined using a riding model, with C—H = 0.93 Å and *U*_iso_(H) = 1.2 *U*_eq_ (parent atom). H atoms involved in hydrogen bonds were located from differential fourier map and refined isotropically.

### Crystal data

**Table:**

C_6_H_7_N_2_O_2_^+^·Br^−^	*F*(000) = 864
*M**_r_* = 219.05	*D*_x_ = 1.876 Mg m^−^^3^*D*_m_ = 1.86 (1) Mg m^−^^3^*D*_m_ measured by Flotation technique using a liquid-mixture of carbon tetrachloride and bromoform
Orthorhombic, *P**b**c**a*	Mo *K*α radiation, λ = 0.71073 Å
Hall symbol: -P 2ac 2ab	Cell parameters from 2610 reflections
*a* = 8.0268 (8) Å	θ = 2.6–24.6°
*b* = 8.1242 (7) Å	µ = 5.25 mm^−^^1^
*c* = 23.7912 (19) Å	*T* = 293 K
*V* = 1551.5 (2) Å^3^	Block, colourless
*Z* = 8	0.21 × 0.19 × 0.17 mm

### Data collection

**Table:**

Bruker SMART APEX CCD area-detector diffractometer	1107 reflections with *I* > 2σ(*I*)
Radiation source: fine-focus sealed tube	*R*_int_ = 0.085
graphite	θ_max_ = 25.0°, θ_min_ = 1.7°
ω scans	*h* = −9→9
12000 measured reflections	*k* = −9→9
1372 independent reflections	*l* = −28→28

### Refinement

**Table:**

Refinement on *F*^2^	Primary atom site location: structure-invariant direct methods
Least-squares matrix: full	Secondary atom site location: difference Fourier map
*R*[*F*^2^ > 2σ(*F*^2^)] = 0.031	Hydrogen site location: inferred from neighbouring sites
*wR*(*F*^2^) = 0.076	H atoms treated by a mixture of independent and constrained refinement
*S* = 0.96	*w* = 1/[σ^2^(*F*_o_^2^) + (0.046*P*)^2^] where *P* = (*F*_o_^2^ + 2*F*_c_^2^)/3
1372 reflections	(Δ/σ)_max_ = 0.001
112 parameters	Δρ_max_ = 0.74 e Å^−^^3^
0 restraints	Δρ_min_ = −0.38 e Å^−^^3^

### Special details

**Table:**

Geometry. All e.s.d.'s (except the e.s.d. in the dihedral angle between two l.s. planes) are estimated using the full covariance matrix. The cell e.s.d.'s are taken into account individually in the estimation of e.s.d.'s in distances, angles and torsion angles; correlations between e.s.d.'s in cell parameters are only used when they are defined by crystal symmetry. An approximate (isotropic) treatment of cell e.s.d.'s is used for estimating e.s.d.'s involving l.s. planes.
Refinement. Refinement of *F*^2^ against ALL reflections. The weighted *R*-factor *wR* and goodness of fit *S* are based on *F*^2^, conventional *R*-factors *R* are based on *F*, with *F* set to zero for negative *F*^2^. The threshold expression of *F*^2^ > σ(*F*^2^) is used only for calculating *R*-factors(gt) *etc*. and is not relevant to the choice of reflections for refinement. *R*-factors based on *F*^2^ are statistically about twice as large as those based on *F*, and *R*- factors based on ALL data will be even larger.

### Fractional atomic coordinates and isotropic or equivalent isotropic displacement parameters (Å^2^)

**Table:**

	*x*	*y*	*z*	*U*_iso_*/*U*_eq_	
Br1	0.79124 (4)	0.19991 (4)	0.286284 (13)	0.04040 (17)	
C1	0.8664 (4)	0.5387 (3)	0.09113 (11)	0.0305 (7)	
C2	0.8275 (3)	0.4633 (3)	0.14194 (11)	0.0282 (7)	
C3	0.6626 (4)	0.4451 (4)	0.15682 (13)	0.0348 (7)	
H3	0.6347	0.3967	0.1910	0.042*	
C4	0.5389 (4)	0.4989 (5)	0.12095 (13)	0.0432 (8)	
H4	0.4276	0.4834	0.1305	0.052*	
C5	0.5790 (4)	0.5754 (4)	0.07105 (13)	0.0462 (9)	
H5	0.4948	0.6128	0.0474	0.055*	
C6	0.7426 (4)	0.5965 (4)	0.05606 (13)	0.0410 (8)	
H6	0.7698	0.6493	0.0226	0.049*	
N2	0.9543 (4)	0.4115 (4)	0.18231 (12)	0.0329 (6)	
N1	1.0379 (4)	0.5526 (3)	0.07159 (10)	0.0399 (7)	
O1	1.1385 (3)	0.4512 (3)	0.08824 (9)	0.0533 (7)	
O2	1.0721 (3)	0.6593 (3)	0.03787 (11)	0.0675 (8)	
H1N	1.020 (4)	0.348 (4)	0.1700 (14)	0.040 (11)*	
H2N	1.020 (7)	0.494 (6)	0.1982 (18)	0.100 (16)*	
H3N	0.902 (6)	0.351 (5)	0.2156 (15)	0.070 (12)*	

### Atomic displacement parameters (Å^2^)

**Table:**

	*U*^11^	*U*^22^	*U*^33^	*U*^12^	*U*^13^	*U*^23^
Br1	0.0387 (2)	0.0383 (2)	0.0443 (3)	0.00825 (15)	0.00844 (14)	0.00908 (14)
C1	0.0328 (16)	0.0321 (17)	0.0266 (16)	0.0011 (14)	0.0013 (13)	−0.0034 (12)
C2	0.0276 (15)	0.0292 (16)	0.0277 (16)	0.0021 (12)	−0.0041 (12)	−0.0039 (12)
C3	0.0304 (16)	0.0439 (19)	0.0300 (16)	−0.0013 (15)	0.0029 (13)	−0.0014 (14)
C4	0.0295 (17)	0.058 (2)	0.0422 (19)	0.0049 (17)	−0.0016 (15)	−0.0075 (17)
C5	0.041 (2)	0.058 (2)	0.0395 (18)	0.0158 (17)	−0.0142 (15)	−0.0036 (17)
C6	0.050 (2)	0.0449 (19)	0.0280 (17)	0.0063 (17)	−0.0044 (14)	0.0035 (14)
N2	0.0288 (15)	0.0392 (16)	0.0306 (15)	−0.0038 (15)	−0.0048 (12)	0.0051 (14)
N1	0.0394 (16)	0.0507 (18)	0.0296 (14)	−0.0036 (15)	0.0023 (12)	−0.0026 (13)
O1	0.0339 (13)	0.0780 (19)	0.0481 (14)	0.0083 (13)	0.0047 (11)	0.0051 (13)
O2	0.0715 (19)	0.0676 (16)	0.0635 (17)	−0.0107 (16)	0.0210 (14)	0.0255 (14)

### Geometric parameters (Å, °)

**Table:**

C1—C6	1.380 (4)	C5—C6	1.372 (5)
C1—C2	1.391 (4)	C5—H5	0.9300
C1—N1	1.458 (4)	C6—H6	0.9300
C2—C3	1.378 (4)	N2—H1N	0.79 (3)
C2—N2	1.461 (4)	N2—H2N	0.93 (6)
C3—C4	1.380 (4)	N2—H3N	1.02 (4)
C3—H3	0.9300	N1—O2	1.213 (3)
C4—C5	1.378 (5)	N1—O1	1.220 (3)
C4—H4	0.9300		
			
C6—C1—C2	120.9 (3)	C4—C5—H5	119.9
C6—C1—N1	117.5 (3)	C5—C6—C1	119.3 (3)
C2—C1—N1	121.6 (3)	C5—C6—H6	120.3
C3—C2—C1	119.1 (3)	C1—C6—H6	120.3
C3—C2—N2	118.0 (3)	C2—N2—H1N	114 (2)
C1—C2—N2	122.8 (3)	C2—N2—H2N	117 (3)
C2—C3—C4	119.9 (3)	H1N—N2—H2N	103 (4)
C2—C3—H3	120.1	C2—N2—H3N	111 (2)
C4—C3—H3	120.1	H1N—N2—H3N	104 (3)
C5—C4—C3	120.5 (3)	H2N—N2—H3N	106 (3)
C5—C4—H4	119.8	O2—N1—O1	123.2 (3)
C3—C4—H4	119.8	O2—N1—C1	118.7 (3)
C6—C5—C4	120.3 (3)	O1—N1—C1	118.0 (3)
C6—C5—H5	119.9		
			
C6—C1—C2—C3	0.7 (4)	C4—C5—C6—C1	0.7 (5)
N1—C1—C2—C3	−176.3 (3)	C2—C1—C6—C5	−1.6 (5)
C6—C1—C2—N2	−175.3 (3)	N1—C1—C6—C5	175.5 (3)
N1—C1—C2—N2	7.8 (4)	C6—C1—N1—O2	25.6 (4)
C1—C2—C3—C4	1.1 (5)	C2—C1—N1—O2	−157.3 (3)
N2—C2—C3—C4	177.3 (3)	C6—C1—N1—O1	−151.2 (3)
C2—C3—C4—C5	−2.0 (5)	C2—C1—N1—O1	25.9 (4)
C3—C4—C5—C6	1.1 (5)		

### Hydrogen-bond geometry (Å, °)

**Table:**

*D*—H···*A*	*D*—H	H···*A*	*D*···*A*	*D*—H···*A*
N2—H1N···O1	0.79 (3)	2.32 (3)	2.702 (4)	111 (3)
N2—H1N···Br1^i^	0.79 (3)	2.70 (4)	3.291 (3)	133 (3)
N2—H2N···Br1^ii^	0.93 (6)	2.29 (6)	3.197 (3)	165 (4)
N2—H3N···Br1	1.02 (4)	2.26 (4)	3.284 (3)	176 (3)

Symmetry codes: (i) *x*+1/2, *y*, −*z*+1/2; (ii) −*x*+2, *y*+1/2, −*z*+1/2.
